# Does Family History of Obesity, Cardiovascular, and Metabolic Diseases Influence Onset and Severity of Childhood Obesity?

**DOI:** 10.3389/fendo.2018.00187

**Published:** 2018-05-02

**Authors:** Domenico Corica, Tommaso Aversa, Mariella Valenzise, Maria Francesca Messina, Angela Alibrandi, Filippo De Luca, Malgorzata Wasniewska

**Affiliations:** ^1^Department of Human Pathology of Adulthood and Childhood, Unit of Pediatrics, University of Messina, Messina, Italy; ^2^Department of Economics, Unit of Statistical and Mathematical Science, University of Messina, Messina, Italy

**Keywords:** childhood obesity, parental obesity, insulin resistance, cardiometabolic risk, body mass index

## Abstract

**Objectives:**

The objectives were to evaluate (1) the metabolic profile and cardiometabolic risk in overweight/obese children at first assessment, stratifying patients according to severity of overweight and age; and (2) to investigate the relationship between family history (FH) for obesity and cardiometabolic diseases and severity of childhood obesity.

**Methods:**

In this cross-sectional, retrospective, observational study, 260 children (139 female), aged between 2.4 and 17.2 years, with overweight and obesity were recruited. Data regarding FH for obesity and cardiometabolic diseases were collected. Each patient underwent clinical and auxological examination and fasting blood sampling for metabolic profile. Homeostasis model assessment of insulin resistance (HOMA-IR), triglyceride-to-high-density lipoprotein cholesterol ratio, and atherogenic index of plasma were calculated. To evaluate the severity of obesity, children were divided into two groups for BMI standard deviation (SD) ≤2.5 and BMI SD >2.5. Moreover, study population was analyzed, dividing it into three groups based on the chronological age of patient (<8, 8–11, >11 years).

**Results:**

BMI SD was negatively correlated with chronological age (*p* < 0.005) and significantly higher in the group of children <8 years. BMI SD was positively associated with FH for obesity. Patients with more severe obesity (BMI SD >2.5) were younger (*p* < 0.005), mostly prepubertal, presented a significantly higher HOMA-IR (*p* = 0.04), and had a significantly higher prevalence of FH for arterial hypertension, type 2 diabetes mellitus, and coronary heart disease than the other group.

**Conclusion:**

(1) Family history of obesity and cardiometabolic diseases are important risk factors for precocious obesity onset in childhood and are related to the severity of obesity. (2) Metabolic profile, especially HOMA-IR, is altered even among the youngest obese children at first evaluation. (3) Stratification of obesity severity, using BMI SD, is effective to estimate the cardiometabolic risk of patients.

## Introduction

Childhood obesity (ChO) is one of the major public health issues worldwide and is known to be associated with an increased risk of severe long-term complications in adulthood (1–3). Obese children have a greater risk of developing arterial hypertension (AH), dyslipidemia, impaired glucose tolerance, insulin resistance (IR) or type 2 diabetes mellitus (T2DM), and precocious atherosclerosis (4–6). Long-term prediction of cardiometabolic risk is strictly conditioned by the severity of obesity (7).

BMI has been correlated with total body fat and cardiovascular risk factors (8); therefore, it would appear useful to evaluate the degree of obesity in the clinical setting. Furthermore, several metabolic and inflammatory factors are involved in the pathogenesis of cardiovascular and metabolic risk in obese children. IR is an important link between obesity and associated cardiovascular and metabolic risk (9). Triglyceride-to-high-density lipoprotein (HDL) cholesterol ratio (TG/HDL ratio) (10) and the atherogenic index of plasma (AIP) (11) may be associated to cardiovascular risk in ChO; however, only few papers have evaluated these parameters in children.

Aims of our cross-sectional, retrospective, observational study were (1) to evaluate, at first assessment, metabolic profile and cardiometabolic risk in overweight/obese children, stratifying them according to the severity of overweight and age; (2) to investigate the relationship between family history (FH) for obesity and cardiovascular and metabolic diseases and severity of ChO.

## Materials and Methods

### Subjects

Two hundred and sixty overweight and obese children and adolescents (139 female), aged between 2.4 and 17.2 years, referred by their family pediatrician, were admitted to the Pediatric Endocrinology Outpatient Clinic at the University of Messina, from January 2010 to December 2013. Analysis of data was carried out retrospectively and completely anonymously. Retrospective analysis of data was notified to the Ethics Committee.

The inclusion criteria for the present study were chronological age between 2 and 18 years, BMI SD >2 from 2 to 5 years (12), and BMI SD >1 over 5 years of age corrected for sex (13), born as healthy full-term infant adequate for gestational age, blood pressure within normal range corrected for age. Exclusion criteria were genetic and endocrinological causes of obesity, born as preterm or postterm, diagnosis of chronic diseases, chronic therapies, and smoking.

### Methods

Detailed history from the parents and from clinical records and family pediatrician data was obtained. Data on FH for obesity, AH, T2DM, dyslipidemia, coronary heart disease (CHD) in parents, siblings, and grandparents were also collected. The following measurements were obtained with standard methods: height, weight, and BMI. Body weight was determined to the nearest 0.1 kg on accurate and properly calibrated standard beam scales, in minimal underclothes and no shoes. Height was measured to the nearest 0.1 cm on standardized, wall-mounted height boards, according to standardized procedures. The children stood with the head aligned in the Frankfort plane, barefoot, with feet placed together and flat on the ground, heels, buttocks, and scapulae against the vertical backboard, arms loose and relaxed with the palms facing medially. BMI was calculated using the equation: body weight (kg)/height (m)^2^. We considered BMI >2.5 SD (>99th percentile corrected for age and sex) to define severe obesity (14). The subjects underwent a detailed physical examination and pubertal evaluation, assessed by five Tanner stages of breast development in girls and testicular volume in boys (15), performed by pediatric endocrinologists. A fasting blood sampling for plasma triglycerides, HDL, low-density lipoproteins (LDL), total cholesterol, glucose, and insulin was performed at least 8 h after the last meal. These parameters were analyzed with standard techniques: triglycerides were measured enzymatically, the HDL-cholesterol fraction was obtained after precipitation using a phosphotungistic reagent, glucose was measured using a glucose oxidase method; serum insulin was determined by a chemiluminescence immunoassay. We considered abnormal levels of triglycerides, total cholesterol, LDL, HDL as defined by the National Cholesterol Education Panel (14): total cholesterol >170 mg/dl, LDL >130 mg/dl, HDL <40 mg/dl, triglycerides >110 mg/dl. We considered abnormal fasting glucose >100 mg/dl and fasting insulin >15 μUI/ml. IR was measured through homeostasis model assessment of insulin resistance (HOMA-IR). This index was calculated using the equation: fasting insulin (μU/ml) × fasting glucose (mg/dl)/405 (16). AIP was calculated using the equation: [LOG (triglycerides/HDL cholesterol)] (17). We considered the following cutoffs to define the three indices of cardiometabolic risk as abnormal: HOMA-IR >2.5 (16), AIP >0.11 (17), and TG/HDL ratio >1.25 (18). This study was carried out in accordance with the World Medical Association’s Declaration of Helsinki.

### Statistical Analysis

Numerical data are expressed as mean and standard deviation (SD) and categorical variables as numbers and percentages. Most of the examined variables were normally distributed as verified by Kolmogorov–Smirnov test; consequently, the parametric approach was used. In order to compare the two groups (BMI SD ≤2.5 vs BMI SD >2.5) with reference to categorical variables, the chi-squared test was applied; with reference to numerical parameters, Student’s *t*-test was estimated. Some univariate linear regression models were estimated to assess the possible dependence of BMI SD on some potential explicative variables, such as chronological age, sex, FH for obesity, T2DM, AH, dyslipidemia, CHD, total cholesterol, LDL, HDL, HOMA-IR, AIP, or TG/HDL ratio. A stepwise multivariate regression model was estimated to identify the most significant predictors of BMI SD. To evaluate the distribution of BMI SD, according to familial risk categories, Student’s *t*-test was estimated. Some univariate logistic regression models were estimated to verify the possible dependence of dichotomous variables, such as HOMA-IR (<2.5 and ≥2.5), AIP (<0.11 and ≥0.11), and TG/HDL ratio (<1.25 and ≥1.25), on some potential explicative variables such as age, sex, FH for obesity, T2DM, AH, dyslipidemia, CHD, total cholesterol, LDL, HDL, and HOMA-IR, AIP, or TG/HDL ratio, respectively. For each parameter, a stepwise multivariate logistic regression model was estimated to identify the most significant predictors.

Furthermore, the whole population was stratified into three groups based on age of patients, and numerically balanced: group 1 (<8 years), group 2 (8–11 years), and group 3 (>11 years). Differences among these strata were evaluated by ANOVA (for numerical parameters) and chi-squared test (for categorical variables). For only variables that significantly differ in comparison among three groups, the pairwise comparisons were performed, using Student’s *t*-test (for numerical parameters) and chi-squared test (for categorical variables); in this context, we applied the Bonferroni correction to control the multiplicity effect.

Statistical analyses were performed using SPSS 17.0 for Window package. *P* < 0.050 two-sided was considered to be statistically significant.

## Results

The mean age of study participants was 9.2 ± 2.9 years. One hundred and thirty-nine subjects (53.4%) were female. One hundred and sixty-one subjects (61.9%) were prepubertal. FH for obesity, T2DM, AH, dyslipidemia, CHD in parents, siblings, and grandparents was positive in 196 subjects (75.4%). The mean values of BMI and BMI SD were 29.7 ± 4.6 and 2.6 ± 0.5 SD, respectively.

### Laboratory Parameters

Total cholesterol >170 mg/dl was seen in 105 patients (40.4%), and in 29 subjects (11.2%), it was >200 mg/dl. LDL was >130 mg/dl in 30 subjects (11.5%); triglycerides were >110 mg/dl in 37 children (14.2%). HDL was <40 mg/dl in 40 children (15.3%). The mean value of TG/HDL ratio was 1.7 ± 0.84. TG/HDL ratio was >1.25 in 166 subjects (63.8%). The mean value of AIP was −0.18 ± 0.2; AIP was >0.11 only in 14 patients (5.4%). Twenty-nine patients had fasting blood glucose >100 mg/dl; those subjects underwent an oral glucose tolerance test that excluded diabetes and confirmed a condition of impaired glucose tolerance. Fasting insulin was >15 μUI/ml in 88 children (33.8%). None of the patients received a diagnosis of metabolic syndrome according to Weiss et al. criteria (19). The mean value of HOMA-IR was 3 ± 2.1. Considering children divided for HOMA-IR <2.5 or ≥2.5, HOMA-IR ≥2.5 was positively correlated with age of patients (*p* < 0.05), triglycerides (*p* < 0.05), AIP (*p* < 0.05), and TG/HDL ratio (*p* = 0.02).

### Evaluation According to BMI SD

BMI SD was negatively correlated with age of patients (*p* < 0.005). A negative association between BMI SD and age of patient was confirmed by univariate linear regression analysis (*p* < 0.005) and by stepwise multivariate regression analysis (*p* < 0.005). Moreover, a positive association between BMI SD and FH for obesity was demonstrated by univariate linear regression analysis (*p* = 0.002) and by stepwise multivariate regression analysis (*p* = 0.007).

Furthermore, the evaluation of BMI SD distribution according to familial risk categories confirmed a significantly higher BMI SD among children with a positive FH for obesity and for dyslipidemia (Table 1).

**Table 1:** BMI SD distribution (mean ± SD) in children according to familial risk factors.

	Positive FH	Negative FH	*p*-value
FH for obesity	2.7 ± 0.5	2.5 ± 0.4	*0.005*
FH for T2DM	2.6 ± 0.3	2.5 ± 0.5	n.s.
FH for AH	2.6 ± 0.4	2.5 ± 0.5	n.s.
FH for CHD	2.6 ± 0.4	2.6 ± 0.5	n.s.
FH for dyslipidemia	2.7 ± 0.6	2.5 ± 0.4	*0.013*

*n.s., not statistically significant; FH, familiar history; AH, arterial hypertension; CHD, coronary heart disease; T2DM, type 2 diabetes mellitus*.

To evaluate FH, clinical characteristics, and metabolic profile according to the severity of obesity, children were divided into two groups with BMI SD ranging from >2 to ≤2.5 (group A, median 2.3) and from >2.5 to 5.2 (group B, median 2.7), respectively. Detailed results concerning the two groups are presented in Table 2. Patients of group B were significantly younger than of group A, and most of them were prepubertal. Furthermore, children of group B had a significantly higher positive FH for T2DM, AH, and CHD and a significantly higher mean value of fasting insulin and HOMA-IR compared to those of group A. Other parameters did not present significant differences between groups (Table 2). Univariate logistic regression analysis showed a negative association between BMI SD >2.5 and age of patients (*p* < 0.005), and a positive association among BMI SD >2.5 and FH for AH (*p* = 0.02), FH for T2DM (*p* = 0.003), and HOMA-IR (*p* = 0.046). These associations were confirmed by stepwise multivariate logistic regression analysis (Table 3).

**Table 2:** Family history, clinical, and biochemical data according to BMI SD ≤2.5 or >2.5.

	Group A BMI SD ≤2.5 (*n* = 126) (mean ± SD)	Group B BMI SD >2.5 (*n* = 134) (mean ± SD)	*p*-Value
Age (years)	10.5 ± 2.3	7.9 ± 2.8	*<0.0005*
Gender (M/F)	63/63	58/76	n.s.
Prepubertal/pubertal	63/63	98/36	*<0.0005*
FH for obesity (yes/no)	37/89	53/81	n.s.
FH for AH (yes/no)	46/80	68/66	*0.02*
FH for T2DM (yes/no)	47/79	75/59	*0.003*
FH for dyslipidemia (yes/no)	26/97	43/91	n.s.
FH for CHD (yes/no)	14/112	27/107	*0.04*
Fasting insulin (μUI/ml)	12.8 ± 8.7	15 ± 9.3	*0.04*
Fasting glucose (mg/dl)	87.4 ± 9.2	87.7 ± 13.4	n.s.
Total cholesterol (mg/dl)	166.7 ± 34.1	162.5 ± 30.7	n.s.
HDL (mg/dl)	50.9 ± 9.7	49 ± 9.3	n.s.
LDL (mg/dl)	99.7 ± 30.7	97.6 ± 26.2	n.s.
Triglycerides (mg/dl)	79.1 ± 41.8	79.7 ± 28.5	n.s.
HOMA-IR	2.8 ± 1.9	3.3 ± 2.3	0.04
AIP	−0.2 ± 0.2	−0.2 ± 0.2	n.s.
TG/HDL ratio	1.6 ± 0.9	1.7 ± 0.7	n.s.

*Differences among groups were evaluated by Student’s t-test (for numerical parameters) and chi-squared test (for categorical variables: gender, pubertal stages, and FH)*.

*“Yes/No” refer to the number of patients*.

*Male (M), Female (F), not statistically significant (n.s.), familiar history (FH), arterial hypertension (AH), coronary heart disease (CHD), type 2 diabetes mellitus (T2DM), homeostasis model assessment of insulin resistance (HOMA-IR), triglyceride-to-HDL cholesterol ratio (TG/HDL ratio), atherogenic index of plasma (AIP), low-density lipoprotein (LDL), high-density lipoprotein (HDL)*.

**Table 3:** Stepwise multivariate logistic regression analysis for BMI SD, HOMA-IR, TG/HDL ratio, and AIP.

Predictors	*B*	Odds ratio (CI 95%)	*p*-Value
**BMI *SD ≤2.5 or >2.5***			
Age	−0.487	0.615 (0.536–0.705)	<0.005
FH for T2DM	0.710	2.034 (1.123–3.684)	0.019
FH for AH	0.738	2.091 (1.142–3.829)	0.017
HOMA-IR	0.284	1.328 (1.114–1.583)	0.002
**HOMA-IR *<2.5 or ≥2.5***			
Age	0.25	1.292 (1.13–1.47)	<0.005
Sex (F)	−0.922	0.39 (0.23–0.68)	0.001
BMI SD	0.87	2.39 (1.15–4.97)	0.01
**TG/HDL ratio *<1.25 or ≥1.25***			
Age	0.289	1.335 (1.153–1.154)	<0.005
Sex (F)	−0.992	0.371 (0.207–0.664)	0.001
BMI SD	1.171	3.224 (1.394–7.456)	0.006
LDL	0.018	1.019 (81.008–1.029)	0.001
**AIP *<0.11 or ≥0.11***			
Total cholesterol	0.05	1.053 (1.006–1.101)	0.02

*Confidence interval (CI), Arterial hypertension (AH), coronary heart disease (CHD), type 2 diabetes mellitus (T2DM), Homeostasis model assessment of insulin resistance (HOMA-IR), triglyceride-to-HDL cholesterol ratio (TG/HDL ratio), atherogenic index of plasma (AIP), low-density lipoprotein (LDL), Female (F)*.

### Evaluation According to Age Subgroups

To verify the precocious incidence of obesity and metabolic complications related to obesity, we divided our population into three groups, numerically balanced, according to age of patients: *group 1* (<8 years; 86 subjects), *group 2* (8–11 years; 102 subjects), *group 3* (>11 years; 72 subjects). In Table 4, the results obtained from group comparisons for both categorical variables and numerical parameters are reported. The pairwise comparisons demonstrated significant differences in BMI SD and BMI SD >2.5, and in fasting insulin, HOMA-IR, and HOMA-IR ≥2.5, reported in Table 5.

**Table 4:** Comparison among three groups according to age of patients.

	Group 1 <8 years (*n* = 86)	Group 2 8–11 years (*n* = 102)	Group 3 >11 years (*n* = 72)	*p*-Value
Age (years)	6.07 ± 1.3	9.4 ± 0.86	12.7 ± 1.6	<*0.05*
Gender (F/M)	57/29	52/50	31/41	<*0.05*
Prepubertal/pubertal	81/5	59/43	21/51	<*0.05*
FH for obesity (yes/no)	35/51	35/67	20/52	n.s.
FH for AH (yes/no)	38/48	44/58	32/40	n.s.
FH for T2DM (yes/no)	43/43	51/51	28/44	n.s.
FH for dyslipidemia (yes/no)	27/59	28/74	17/55	n.s.
FH for CHD (yes/no)	11/75	15/87	15/57	n.s.
BMI SD	2.9 ± 0.5	2.4 ± 0.2	2.3 ± 0.3	<*0.05*
HOMA-IR	2.8 ± 2.1	2.7 ± 1.5	3.6 ± 2.5	<*0.05*
Fasting insulin (μUI/ml)	13.1 ± 2.1	12.9 ± 6.8	16.4 ± 11.3	<*0.05*
Fasting glucose (mg/dl)	86.7 ± 9.8	87.3 ± 9.9	88.9 ± 14.9	n.s
Total cholesterol (mg/dl)	164.7 ± 29.5	167.3 ± 36.5	160.6 ± 29.1	n.s.
HDL (mg/dl)	50.3 ± 9.7	49.9 ± 9.4	49.5 ± 9.5	n.s.
LDL (mg/dl)	98.9 ± 26.9	101.2 ± 31.8	94.5 ± 25	n.s.
Triglycerides (mg/dl)	75.5 ± 27.6	80.3 ± 36.7	82.7 ± 42	n.s.
AIP	−0.19 ± 0.2	−0.17 ± 0.19	−0.16 ± 0.2	n.s.
TG/HDL ratio	1.5 ± 0.7	1.7 ± 0.8	1.7 ± 0.9	n.s.
BMI SD >2.5 (yes/no)	73/13	38/64	23/49	<*0.05*
HOMA-IR >2.5 (yes/no)	41/45	46/56	47/25	<*0.05*
AIP >0.11 (yes/no)	6/80	5/97	3/69	n.s.
TG/HDL ratio >1.25 (yes/no)	50/36	65/37	51/21	n.s.

*Differences among groups were evaluated by ANOVA (for numerical parameters) and chi-squared test (for categorical variables: gender, FH, BMI SD >2.5, HOMA-IR >2.5, AIP >0.11, TG/HDL ratio >1.25). “Yes/No” refer to the number of patients*.

*Male (M), Female (F), not statistically significant (n.s.), arterial hypertension (AH), type 2 diabetes mellitus (T2DM), coronary heart disease (CHD), homeostasis model assessment of insulin resistance (HOMA-IR), triglyceride-to-HDL cholesterol ratio (TG/HDL ratio), atherogenic index of plasma (AIP), low-density lipoprotein (LDL), high-density lipoprotein (HDL)*.

**Table 5:** Pairwise comparisons among three groups according to age of patients.

	*p*-Value Group 1 vs 2	*p*-Value Group 1 vs 3	*p*-Value Group 2 vs 3
BMI SD	<0.001	<0.001	n.s.
HOMA-IR	n.s.	0.006	0.006
Fasting insulin (µUI/ml)	n.s.	0.007	0.006
BMI SD >2.5	<0.001	<0.001	n.s.
HOMA-IR >2.5	n.s.	n.s.	0.009

*For only variables that significantly differ in comparison among three groups (Table 4), the pairwise comparisons were performed, using Student’s *t*-test (for numerical parameters) and chi-squared test (for categorical variables); in this context, we applied the Bonferroni correction to control the multiplicity effect (adjusted α is 0.017)*.

## Discussion

In our observational, cross-sectional, retrospective study, we analyzed a large cohort of children with simple overweight and obesity, included in a wide range of ages, brought to our outpatient clinic for first evaluation.

The main findings of this study are the more severe obesity in younger children and the relationship between FH for obesity, cardiovascular, and metabolic diseases and the severity of obesity. Our findings suggest that the problem of ChO is not only related to an increase in the number of diagnoses among children but also concerns an earlier insurgence of obesity.

Furthermore, we suggest that FH for obesity, AH, T2DM, dyslipidemia, and CHD should be considered risk factors for early onset and a major severity of obesity in children. The findings that FH for obesity significantly increases the risk of ChO have been consistently reported in other studies (20–24). This association is likely the result of a combination of genetic and epigenetic mechanisms and environmental factors, such as shared family lifestyle characteristics.

Our results support the role of FH for obesity, suggesting also the involvement of FH for cardiovascular and metabolic diseases in determining ChO.

In this context, family pediatricians, aware of familiar risk factors for early onset of ChO, play a primary role in identifying children’s overweight in a precocious phase, to start prevention programs. Moreover, although prevention remains the first target in ChO management, pediatricians should focus on the early identification of obesity-associated complications, which may already occur precociously in children.

Early onset of severe obesity increases the risk of long-term obesity, cardiovascular, and metabolic complications (1, 2, 7, 25). Overweight and obesity from childhood to adulthood have been related to an increased risk of T2DM, AH, dyslipidemia, and carotid-artery atherosclerosis. However, it has been reported that in subjects who were overweight or obese in childhood, but had normal weight in adulthood, cardiovascular risk in adulthood is comparable to the general population (4). Therefore, an early, multidisciplinary approach (pediatric, endocrinological, nutritional) in overweight and obese children is necessary to reduce the development of cardiovascular and metabolic complications. IR, previously considered a problem in adulthood, becomes a serious issue also in children. In a cohort of preschool children, Aristizabal et al. reported IR as the most frequent metabolic alteration, supporting the use of BMI to identify children with IR (26). Also in our population, BMI was an efficacious screening method, easy for routine use, to identify children at risk of early obesity complications. IR represents an important link between obesity and associated cardiovascular risk (9) and has been indicated as one of the first mechanisms involved in the development of endothelial dysfunction in obese youths (5). Giannini et al. showed that early changes in glucose metabolism, detected in obese prepubertal children, could be one of the factors leading to the increase of intima media thickness (5). Moreover, IR has been related to the metabolic syndrome incidence in childhood. Weiss et al. demonstrated the association between the severity of obesity and the diagnosis of metabolic syndrome, and the negative effect of obesity worsening on each component of the metabolic syndrome (19). In our cohort, HOMA-IR was abnormal in 51.9% of participants, significantly higher among those with the most severe obesity, despite them being younger and mainly prepubertal.

Furthermore, it is suggested that IR influences the link between obesity and dyslipidemia. Vukovic et al. demonstrated that HOMA-IR was strictly related to lipid profile in their pediatric population (27). Giannini et al. reported a correlation between IR and TG/HDL ratio (5). In our cohort, we demonstrated a correlation between HOMA-IR and lipid profile, in particular with triglycerides, AIP, and TG/HDL ratio. Recently, the importance of TG/HDL ratio as a marker of cardiovascular risk and inflammatory status in children has been demonstrated (10). TG/HDL ratio was frequently abnormal among our patients, even if a significant difference according to the severity of obesity has not been demonstrated. TG/HDL ratio is an easily calculated marker, useful in monitoring obese children at risk of metabolic complications. AIP, considered a new marker of atherogenicity, has been shown to significantly correlate with cardiovascular disease and metabolic syndrome in adulthood (28); however, it has been little studied in children. Vrablik et al. demonstrated the correlation of AIP with BMI and HOMA-IR in children (11). Conversely, we did not confirm these data in our patients, among whom only in few cases was AIP abnormal.

Some limitations of this study need to be acknowledged. In particular, socioeconomic factors have not been considered. Waist circumference was not systematically evaluated during the assessment; therefore, we were not able to estimate the incidence of metabolic syndrome in comparison with other pediatric diagnostic criteria (29), other than Weiss et al. criteria (19). In addition, our cohort of patients comes from southern Italy, which may limit the generalization of results; on the other hand, our population represents a sample with homogeneous features.

To summarize, we suggest the importance of adopting prevention programs to contrast ChO development and, when ChO is already diagnosed, start an early multidisciplinary medical approach in these children, who, even if so young, may already show the first signs of metabolic disorders, possible preluding cardiovascular and metabolic disease development in young adulthood.

We conclude that (1) an FH for obesity, AH, T2DM, or CHD is an important risk factor for precocious obesity onset in childhood and influences the severity of obesity; (2) metabolic profile, especially HOMA-IR, is altered even among the youngest obese children at first evaluation; (3) stratification of the severity of obesity, using BMI SD, is effective to estimate the cardiometabolic risk of patients and to program a specific and multidisciplinary follow-up for each patient.

## Ethics Statement

This study was carried out in accordance with the World Medical Association’s Declaration of Helsinki. Retrospective and anonymous analysis of data was notified to the Ethics Committee.

## Author Contributions

MW and FD conceived the study. TA, MV, and MM contributed to data collection. AA carried out data analysis. DC, MW, TA, and FD carried out data interpretation. DC and MW were involved in literature search and writing of the manuscript. All authors approved the submitted version of the manuscript.

## Conflict of Interest Statement

All the authors have no conflicts of interest relevant to this article to disclose.
